# A hybrid framework of hesitant fuzzy soft sets and rough sets for uncertainty modelling

**DOI:** 10.1038/s41598-025-34368-7

**Published:** 2026-01-02

**Authors:** Jahanvi, Dinesh Kumar Nishad, Rashmi Singh, Saifullah Khalid

**Affiliations:** 1 Department of Mathematics, AIAS, Amity University Uttar Pradesh, Noida, India; 2 Department of Electrical Engineering, Dr. Shakuntala Misra National Rehabilitation University, Lucknow, UP India; 3 IBM Multi Activities Co. Ltd. Khartoum, Khartoum, Sudan

**Keywords:** Hesitant fuzzy soft sets, Rough sets, Covering, Uncertainty modeling, Decision making, Approximation operators, Engineering, Mathematics and computing

## Abstract

The process of decision making involves uncertainty due to lack of agreement among experts, inaccuracy in measurements and incomplete information. Current frameworks are inadequate in dealing with cases in which hesitation, indiscernibility, and parameterization may all take place simultaneously. The article proposes a new Hesitant Fuzzy Soft Rough Set (HFSRS) model that combines hesitant fuzzy soft sets and rough sets with dynamic $\:\boldsymbol{\beta\:}$ covers that changes approximation boundaries in relation to hesitant membership levels. The suggested framework deals with severe constraints such as the impossibility to model parameter-dependent hesitation, duality violation of the classical fuzzy rough sets, and fixed thresholding processes that cannot be used in a noisy environment. The three fundamental properties provided by mathematical formalization: (a) duality preservation to provide logical consistency important for safety-critical applications, (b) monotonicity to provide predictable behavior important to explainable AI systems, and (c) topological consistency to provide hierarchical uncertainty modeling. HFSRS is empirically validated using synthetically generated datasets (500 photovoltaic modules with three fault indicators adjusted to IEC 61215-2:2021 standards) to achieve 92 per cent accuracy versus 85 per cent on classical rough sets, 86 per cent on fuzzy rough sets, 88 per cent on intuitionistic fuzzy rough sets, with 35 per cent reduction in boundary region and AUC of 0.97 versus 0.92 on competing methods running 30 times The best 0.65 -threshold of the beta value balances accuracy and coverage. The HFSRS-TOPSIS algorithm provides practitioners with strong decision support, computational tractability of $\:\left(O\right(n\:\times\:\:m\:\times\:\:k\left)\right)$ on a dataset of up to $\:{10}^{4}$ objects.

## Introduction

Uncertainty is inherent in real-life decision-making. It manifests through incomplete information, conflicting opinions, and measurement errors. There are many dimensions of this complex issue that have been dealt with using traditional approaches, but there are still great gaps in the form of multiple types of uncertainties that come to play simultaneously. The history of uncertainty modelling has unfolded in a series of paradigms, each of which has dealt with the shortcomings of the one before it. It has also added new features to the range of possible ways to deal with the complexity of the real world.

### Historical development and theoretical foundations

This subsection reviews the foundational contributions. Zadeh^1^ proposed the fuzzy sets (FS) to handle the uncertainty situation, considering the element to be a part of a set. This innovative idea permitted mathematical modeling of the unclear concepts and smooth changes between categories. Nevertheless, FS theory presupposes the existing information regarding membership functions which are usually subjective and are hard to define precisely. In addition, it is not sufficient at managing indiscernibility that comes out, because of incomplete or imprecise information. Such constraints were especially apparent when using applications where objective standards are necessary to determine who could be included or excluded or in applications involving granular information structures. To overcome these shortcomings, the rough set (RS) theory presented by Pawlak^2^ offers a special methodology to manage uncertainty and indiscernibility in data. Rough sets compare objects with similar features, unlike FS theory, which employs membership degrees. The objective treatment of uncertainty, with no relevant parameters, subjective judgments were also the focus of the rough set theory, and thus it is particularly suitable to use in the data-driven approach. Even though FS theory and the RS theory address different aspects of uncertainty, both the two theories do not use external parameters, Molodtsov^3^ introduced the concept of soft sets (SS) for addressing uncertainty in DM problems. Soft sets offer a parameterized collection of subsets, without defining membership degrees. SS theory found various real world applications, such as DM algorithm for surgical mask disposal^4^, medical diagnosis^5^, smart systems^6^ etc. Fuzzy soft sets (FSS)^7^ given by Maji et al. combined FS and SS theory for handling uncertainty and then many hybrid structures came into picture^8,9^. Ullah et al.^10^ developed a framework based on three-way DM model under Pythagorean fuzzy credibility numbers and Orhan^11^ extended neutrosophic soft relations to Q-neutrosophic soft relations The theoretical framework has recently been extended to interval-valued bipolar complex FSS^12^, time FSS^13^, fuzzy credibility graphs used in climate mitigation strategy assessment^14^, and concept of nearness in hybrid soft sets^15^.

### The emergence of hesitant fuzzy sets

We next focus on the literature on hesitant fuzzy sets, summarizing key definitions, extensions, and properties that motivate the hesitant fuzzy perspective adopted in this article. Often when a given element has multiple potential membership values, conventional fuzzy methods fail. In real-life DM scenarios, the situation often arises when a group of experts cannot agree on a single value, resulting in hesitation. To fill this gap, hesitant fuzzy sets (HFS) were proposed by Torra^16^. HFS are an extension of FS, but with the ability to have a set of possible membership values of an element. Recently, Lu et al.^17^ improved the HFS structure. This idea was recognized against the backdrop of the fact that decision-makers often struggle to precisely quantify their preferences, especially in scenarios where the criteria are objective or complex. HFS was combined with SS to create hesitant fuzzy soft sets (HFSS), proposed by Babitha and John^18^. Borah and Hazarika^19^ worked on development of HFSS, whereas Vijayalakshmi and Nisha^20^ examined triangular HFSS. The combination is an integration of the parameterization of soft sets with the hesitation-handling of HFS to form a detailed uncertainty model.

### Critical analysis of hybrid framework development

**Fig. 1:**
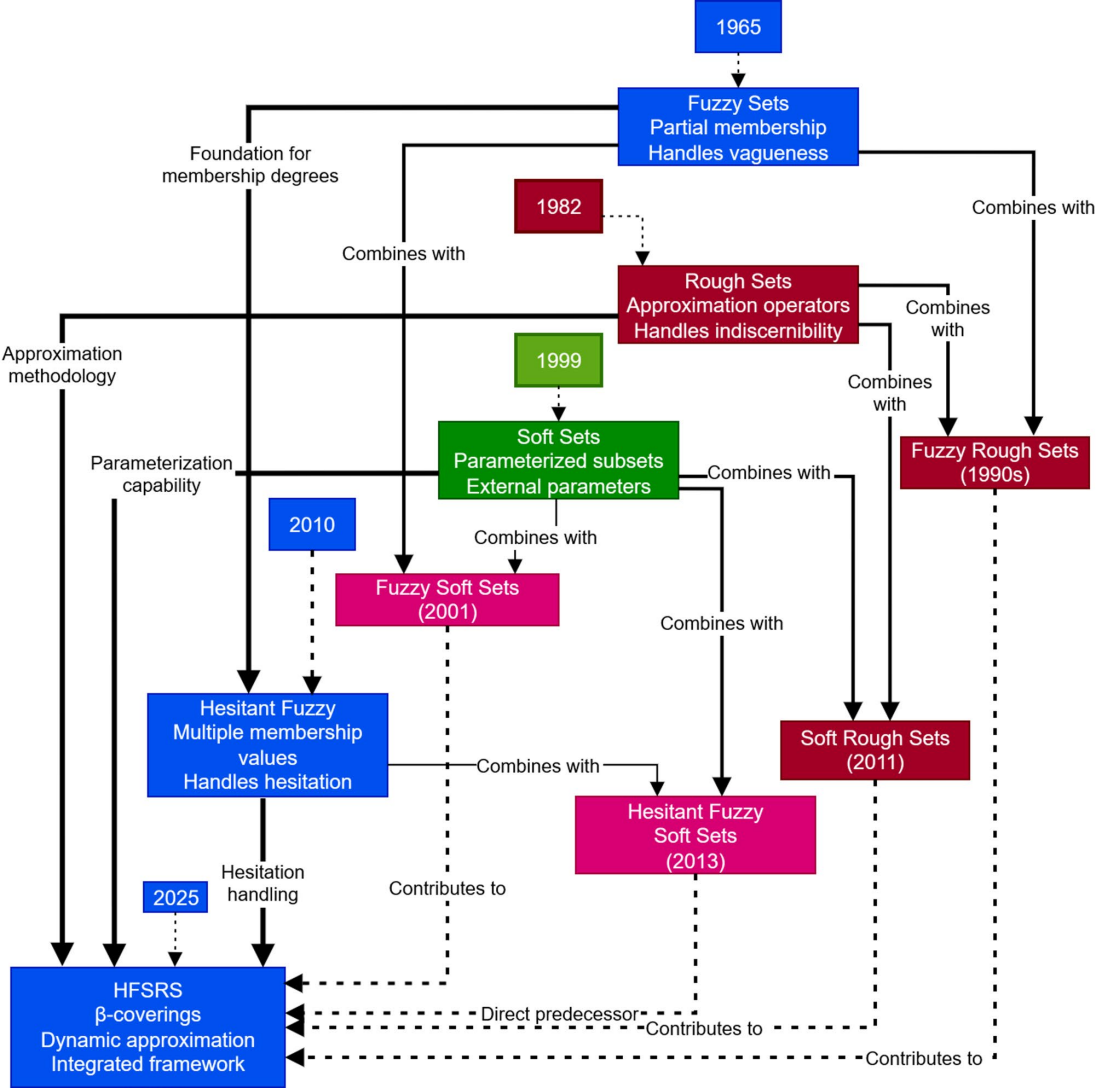
Evolution of uncertainty modelling frameworks.

This subsection critically examines the hybrid frameworks. The logical direction of development in uncertainty modeling has been toward hybrid systems that utilize the strengths of distinct theories. The evolution of frameworks handling uncertainty is illustrated by Fig. 1. Integration of HFS with RS created hesitant fuzzy rough sets (HFRS). HFRS by Zhang et al.^21^, use relations to define approximation operators whereas our framework introduces the first adaptive approximation mechanism for HFSRS. Xie and Gong^22^ presented the hesitant soft fuzzy rough set, which is combination of hesitant, soft, and rough set theories to address the multilayered uncertainty in decision analysis. However dynamic covering and monotonicity were missing. Khan et al.^23^ examined applications of probabilistic hesitant fuzzy rough sets in decision support systems. Study by Mathew et al.^24^ examined multi-granulation picture hesitant fuzzy rough sets enabling decision models to consider information in a multi-perspective granulometry but lack practical validation. Dey et al.^25^ presented a novel approach to hesitant multi-fuzzy SS decision-making operations. Feng et al.^26^ studied three-way DM frameworks based on canonical SS of HFS and showed their suitability for complex decision environments. Attaullah et al.^27^ proposed a DM algorithm to evaluate a wind power plant based on q-rung orthopair hesitant fuzzy rough aggregation information, incorporating the TOPSIS method. In a similar study, Attaullah et al.^28^ proposed fermatean hesitant fuzzy rough aggregation operators, which enhance the strength of the group DM process, but they rely on fixed algebraic aggregation operators and due to rigid cubic membership constraint adaptation to varying levels of data noise, expert hesitancy is limited. Continuing this, Abbas et al.^29^ discussed dual hesitant fuzzy soft rough sets and soft rough dual hesitant FS, creating more modelling frameworks to both upward and downward uncertainty representations but their models use static soft relations without any hesitation dependent adaptation mechanism. Alcantud et al.^30^ developed hesitant fuzzy information-based rough set models to extend the RS theoretical foundation for handling hesitant data but their models are defined on hesitant fuzzy approximation spaces without soft parameterization, so they do not address parameter-dependent hesitation. Albaity et al.^31^ presented a complex hesitant fuzzy rough multi-attribute DM method for effective data source selection.

Zhan et al.^32^ presented a comprehensive survey on three-way behavioral DM with hesitant fuzzy information systems and Zhang et al.^33^ developed multigranulation rough set models. The dual hesitant fuzzy rough set framework^34^ addresses both membership and non-membership hesitancy. Interval-valued hesitant fuzzy multigranulation rough sets have been applied to steam turbine fault diagnosis^35^ and medical diagnoses^36^. Three-way group decisions using multigranulation hesitant fuzzy decision-theoretic rough sets^37^ extend the framework to group scenarios. These developments employ static approximation mechanisms without adaptive threshold-based coverings, which distinguishes our HFSRS framework.

Topological aspects of virtual fuzzy parametrized fuzzy soft sets investigated in^38^, where notions such as closure, interior, and compactness were introduced for hybrid soft structure. In^39^, the authors developed the theory of near soft topological spaces by introducing subspaces and establishing near soft separation axioms $\:{T}_{i}{\hspace{0.25em}\hspace{0.05em}}(0\le\:i\le\:4)$. The work in^40^ proposed a new definition of bipolar soft topology, introduced bipolar soft ordered topology via bipolar soft intervals, and illustrated its applications. Recent studies have explored various aspects of this integration. However, most of the above cited articles lack either adaptive dynamic covering or topological aspects.

### Applications and domain-specific developments

Hybrid structures have been used in many fields to deal with uncertainty have been surveyed in this subsection. A framework based on intuitionistic fuzzy rough sets in two universes by Sun et al.^41^ for decision-making but lack hesitation and parameterization. A forest fire monitoring system was proposed in which Sherstjuk and Zharikova^42^ applied fuzzy rough soft sets to UAV-based recognition. Mishra et al.^43^ employed hesitant fuzzy decision-making models in the selection of drugs for treating mild symptoms of COVID-19. These developments are practically relevant, but none employ adaptive dynamic covering mechanisms. Wider generalizations have also been formulated. Mazarbhuiya and Shenify^44^ employed intuitionistic fuzzy-rough sets to perform specifically anomaly detection tasks. Zhang et al.^45^ introduced an interval-valued hesitant fuzzy multigranulation rough set model for simulation based fault diagnosis of steam turbines.

Hybrid uncertainty frameworks have demonstrated practical relevance across diverse domains. In renewable energy, Ashraf et al.^46^ employed picture HFSS for sustainable solar energy management, while Gupta et al.^47^ developed hesitant bi-fuzzy TOPSIS for renewable energy selection. The fuzzy $\:\beta\:$ covering framework has been extended for attribute reduction and multi-attribute DM^48,49^. Hesitant fuzzy linguistic rough sets address complex decision scenarios^50^. These implementations validate practical utility while highlighting the need for adaptive approximation mechanisms.

### Development of $\:\boldsymbol{\beta\:}$ covering

$\:\beta\:$ covering models introduced to generalize RS theory over fuzzy systems, ensuring greater flexibility in handling uncertainty and vagueness. Ma^51^ universalized RS models with fuzzy lattices and extended the theoretical foundations and practical use to model complex uncertainty. The first designed models were centered on RS of fuzzy $\:\beta\:$ neighborhoods^52^ based on the of rough set approximations. These concepts were implemented in soft set designs, using fuzzy soft $\:\beta\:$ covering approximation spaces introduced^53^, then extended to interval-valued fuzzy soft $\:\beta\:$ covering spaces and HFS $\:\beta\:$ coverings^54^. Together, these models represent a classical set to use in parameterized fuzzy environment, providing potent approximation and decision processes of vagueness.

### Research gaps and motivation

Although much has been done on hybrid models still, systematic review shows that there are four essential gaps in current theories that may achieve a rationale to the current work:

1. As demonstrated by Wang^55^, duality fails in classical fuzzy rough sets under common operators, this fundamental shortcoming remains unaddressed in hesitant soft fuzzy rough sets^22^ which limits reliability of the framework in decision making.
2. Existing models^56^ while dealing with multi source uncertainty often fail to capture complete decision context as they don’t consider probabilistic information associated with Evaluation values alongside there inherent potential uncertainty and risk.
3. Fermatean HFRS by Attaullah et al.^28^ rely on fixed algebraic aggregation operators and due to rigid cubic membership constraint adaption to varying levels of data noise, expert hesitancy is limited.
4. Most models do not have a topological interpretation so that structuring generalization is not possible; nor can the approximation operators be related to topological interior/closure operations which Lashin et al.^57^ propose in the case of classical rough sets.

Conversely, the suggested HFSRS model proposes $\:\beta\:$ adaptive coverings. This provide adaptive approximation limits, and both are duality-preserving operators under hesitant fuzzy structures provide a connection between algebraic consistency and applicable decision situations. Such adaptability and duality makes HFSRS a truly innovative paradigm that cannot be expanded by adding more incremental improvements to the existing hybrid models as demonstrated by Prasertpong^58^, Chang and Wei^59^, and Lu et al.^17^,.

### Novelty and contributions

This study introduces a new HFSRS model with $\:\beta\:$ coverings that dynamically vary the limits of approximations, depending on the degree of hesitancy in membership. Second, we give strict mathematical proofs that the proposed framework maintains its duality, monotonicity, and topological consistency. Third, we generate a standardized HFSRS-TOPSIS algorithm with feasible rules of implementation. Fourth, we provide detailed empirical validation in the photovoltaic fault detection case studies with 92% accuracy and renewable energy technology selection and show that the results have considerable improvements over the traditional method.

### Distinction between adopted foundations and novel contributions

While this study adopts foundational concepts from existing theories specifically HFSS from Babitha and John^18^, rough set approximations from Pawlak^2^, and $\:\beta\:$ covering concepts inspired by recent developments^53,54^ the novelty lies in their integration and extension. Unlike prior works that employ HFSS and RS sequentially, HFSRS introduces dynamic $\:\beta\:$ coverings (Definition 3.1) that adapt approximation boundaries based on hesitant membership degrees, a mechanism absent in existing frameworks. Theorems 3.1–3.4 establish new mathematical properties specific to the HFSRS framework, including duality preservation under hesitant fuzzy operations (Eq. 20) and topological consistency, which are not trivial extensions of prior results. The HFSRS-TOPSIS algorithm (Algorithm 1) gives a novel practical implementation pathway with Einstein aggregation operators (Eqs. 23–25) specifically designed for hesitant fuzzy soft rough approximations. The framework does not merely apply existing theories but synthesizes them into a mathematically rigorous, computationally feasible hybrid model with proven reliability (Table 1: 92% vs. 85–88% for existing methods).

**Table 1:** Domain-specific performance comparison.

Domain	HFSRS Accuracy	Best Competitor	Improvement
Fault Detection	92%	IFS-RS (88%)	+ 4.5%
Energy Selection	94%	Fuzzy RS (87%)	+ 8.0%
Medical Diagnosis	91%	Soft RS (86%)	+ 5.8%
Risk Assessment	93%	Classical RS (84%)	+ 10.7%

The remainder of the paper is structured as follows: Sect. “Historical development and theoretical foundations” will review the fundamentals of the HFSS and RS theory, which will be the theoretical basis of the suggested hybrid framework. Section “The emergence of hesitant fuzzy sets” gives the proposed HFSRS model, the mathematical formulations and proofs. Section “Critical analysis of hybrid framework development” gives algorithmic applications, including case studies. Section “Applications and domain-specific developments” draws a comparative analysis to the existing uncertainty models. Findings are discussed in Sect. Development of covering”, and the future research directions conclude in Sect. “Research gaps and motivation”.

## Preliminaries

The fundamental concepts of HFSS and RS providing theoretical foundation for the proposed hybrid framework are presented in this section.

### Definition 2.1

^18^ Let $\:U$ be a universe of discourse and $\:E$ a set of parameters. A HFSS is a pair $\:\left(F,\:E\right)$, where $\:F:\:E\:\to\:\:P\left(U\right)$ maps each parameter $\:e\:\in\:\:E$ to a subset of $\:U$. For $\:x\:\in\:\:U,\:h_{e}\left(x\right)$, a hesitant fuzzy element (HFE), represents the set of possible membership degrees of $\:x$ under parameter $\:e$.

For two HFSS $\:\left(F,\:A\right)$ and $\:\left(G,\:B\right)$, their union is defined as:

1 $$\:\left(F,A\right)\cup\:\left(G,B\right)=\left(H,C\right)$$

where $\:C\:=\:A\:\cup\:\:B$ and for all $\:e\:\in\:\:C$,

2 $$\:{h}_{e}^{H}\left(x\right)=\left\{\begin{array}{ll}{h}_{e}^{F}\left(x\right)&\:\mathrm{if\:}e\in\:A-B\\\:{h}_{e}^{G}\left(x\right)&\:\mathrm{if\:}e\in\:B-A\\\:{h}_{e}^{F}\left(x\right)\cup\:{h}_{e}^{G}\left(x\right)&\:\mathrm{if\:}e\in\:A\cap\:B\end{array}\right.$$

The intersection $\:\left(F,\:A\right)\cap\:\:\left(G,\:B\right)$ is:

3 $$\:{h}_{e}^{H}\left(x\right)={h}_{e}^{F}\left(x\right)\cap\:{h}_{e}^{G}\left(x\right)$$

The complement $\:{\left(F,A\right)}^{c}$ is:

4 $$\:{h}_{e}^{{F}^{c}}\left(x\right)=1-{h}_{e}^{F}\left(x\right)$$

**Fig. 2:**
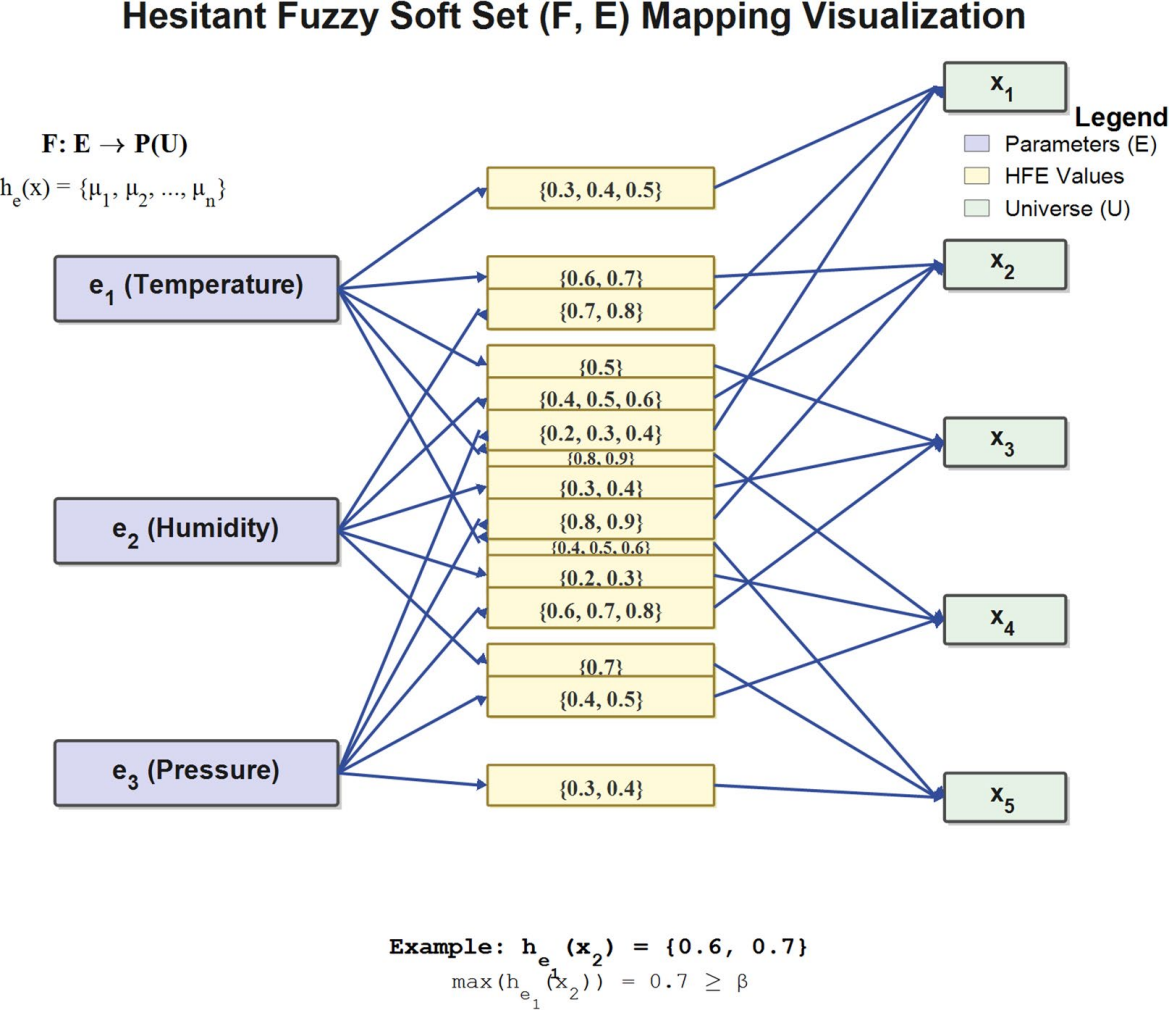
Hesitant fuzzy soft set representation.

Figure 2 illustrates the mapping structure of a hesitant fuzzy soft set, where the parameters $\:E\:=\:\left\{{e}^{1},\:{e}^{2},\:{e}^{3}\right\}$ correspond to hesitant fuzzy elements. For instance,$\:\:{h}^{1}\left({x}^{2}\right)=\:\left\{0.6,\:0.7\right\}$ indicates that under parameter $\:{e}^{1}$ (Temperature), element $\:{x}^{2}$ has two possible membership degrees, capturing the hesitation inherent in expert evaluations.

### Definition 2.2

^2^ Let $\:U$ be a universe and $\:R$ an equivalence relation (indiscernibility relation). The pair $\:\left(U,\:R\right)$ forms an approximation space, partitioning $\:U$ into equivalence classes $\:\left[x\right]_{r}$. For a subset $\:X\:\subseteq\:\:U$, its approximations are defined as:

Lower approximation:

5 $$\:\underset{\_}{R}\left(X\right)=\left\{x\in\:U:{\left[x\right]}_{R}\subseteq\:X\right\}$$

Upper approximation:

6 $$\:\overline{R}\left(X\right)=\left\{x\in\:U:{\left[x\right]}_{R}\cap\:X\ne\:{\varnothing}\right\}$$

Boundary region:

7 $$\:B{N}_{R}\left(X\right)=\overline{R}\left(X\right)-\underset{\_}{R}\left(X\right)$$

Accuracy measure:

8 $$\:{\alpha\:}_{R}\left(X\right)=\frac{\left|\underset{\_}{R}\left(X\right)\right|}{\left|\overline{R}\left(X\right)\right|}$$

**Fig. 3:**
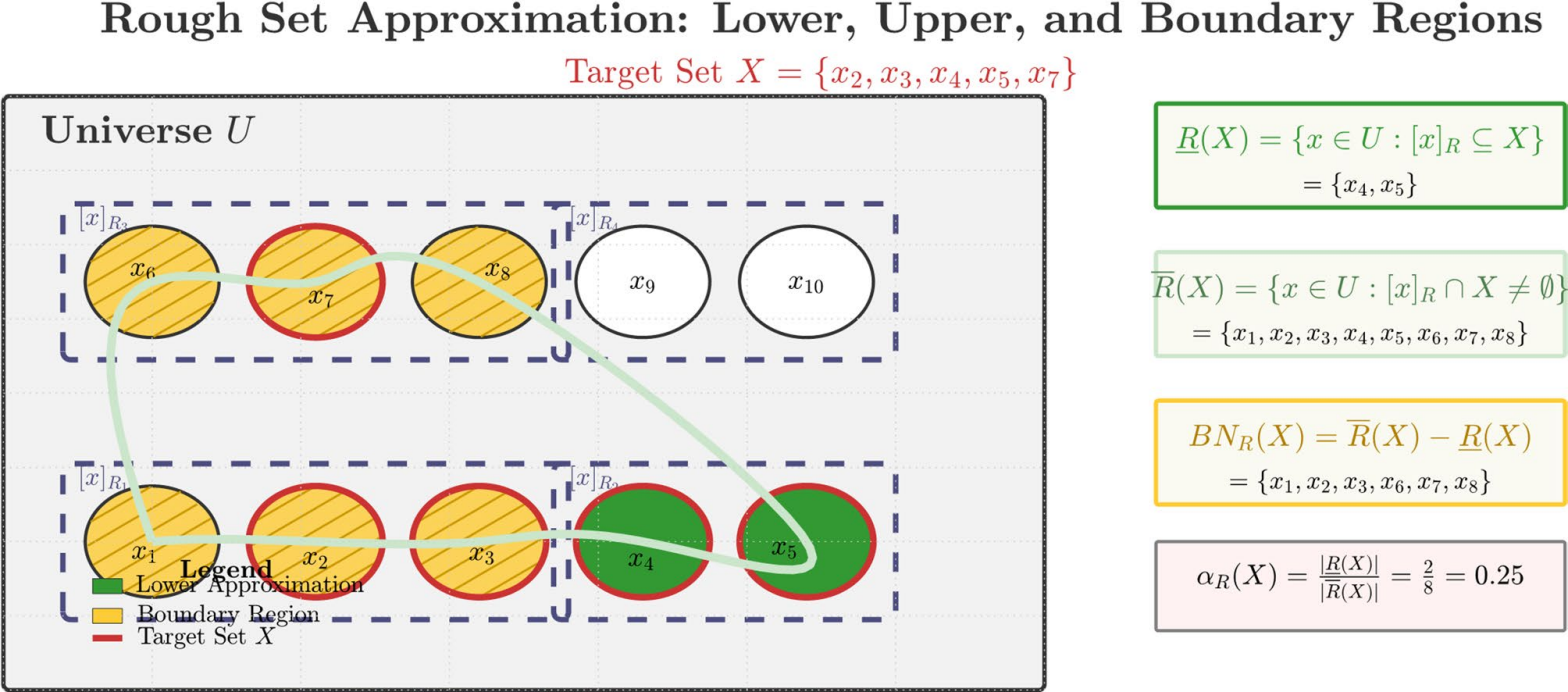
Rough set approximation illustration.

Figure 3 demonstrates the rough set approximation mechanism for a target set $\:X\:=\:\left\{{x}^{2},\:{x}^{3},\:{x}^{4},\:{x}^{5},\:{x}^{7}\right\}$. The lower approximation $\:\bar{R}\left(X\right)$ contains elements whose equivalence classes are entirely within $\:X$, while the upper approximation $\:\bar{R}\left(X\right)$ includes elements whose equivalence classes intersect with $\:X$. The boundary region $\:BN^{R}\left(X\right)$ captures uncertain elements, with the accuracy measure $\:\alpha\:^{\rm R}\left(X\right)=\:0.25$ indicating significant uncertainty in this example.

### Definition 2.3

^60^ Given a weight vector $\:w\:=\:\left({w}^{1},\:{w}^{2},\:\dots\:,\:w_{n}\right)$ for parameters $\:{e}^{1},\:{e}^{2},\:\dots\:,\:e_{n}$, the weighted HFSS aggregates memberships as:

9 $$\:{h}_{agg}\left(x\right)={\oplus{w}_{i}}_{i=1}^{n}\otimes\:{h}_{{e}_{i}}\left(x\right)$$

where ⊕ and ⊗ are appropriate hesitant fuzzy operations.

### Definition 2.4

^60^ The similarity between two HFSS (*F*, *A*) and (*G*, *B*) is computed as:

10 $$\:Sim\left(\left(F,A\right),\left(G,B\right)\right)=\frac{1}{\left|A\cup\:B\right|}\sum\:_{e\in\:A\cup\:B}\frac{\left|{h}_{e}^{F}\left(x\right)\cap\:{h}_{e}^{G}\left(x\right)\right|}{\left|{h}_{e}^{F}\left(x\right)\cup\:{h}_{e}^{G}\left(x\right)\right|}$$

## Hesitant fuzzy soft rough sets (HFSRS)

This section presents the HFSRS framework that addresses the limitations identified in existing uncertainty modeling approaches. The framework incorporates dynamic $\:\beta\:$ coverings that adapt approximation boundaries based on hesitant membership degrees.

### $\:\boldsymbol{\beta\:}$ covering framework

The foundation of HFSRS rests on establishing meaningful coverage relationships. The threshold β allows practitioners to control coverage granularity based on uncertainty tolerance.

#### Definition 3.1

Let $\:U$ be a finite universe, $\:E$ a set of parameters, and $\:\left(F,\:E\right)$ denote a HFSS. Given a threshold $\:\beta\:\:\in\:\:\left[\mathrm{0,1}\right]$, we define the hesitant fuzzy soft $\:\beta\:$ covering as:

11 $$\:{C}_{\beta\:}=\left\{{C}_{e}^{\beta\:}:e\in\:E\right\}$$

where:

12 $$\:{C}_{e}^{\beta\:}=\left\{x\in\:U:\mathrm{m}\mathrm{a}\mathrm{x}\left({h}_{e}\left(x\right)\right)\ge\:\beta\:\right\}$$

This covering ensures that every element $\:x\:\in\:\:U$ is included in at least one subset $\:{C}_{e}^{\beta\:}$ with membership degree exceeding $\:\beta\:$.

#### Remark 3.1

It is noted that this $\:\beta\:$ threshold-based covering is conceptually related to the hesitant fuzzy soft $\:p\beta\:$ covering introduced by Lu^53^ although the present formulation adopts a simplified Max based threshold condition rather than the $\:p$ inclusion operator.

#### Example 3.1

Let $\:U\:=\:\left\{{p}_{1},\:{p}_{2},\:{p}_{3},\:{p}_{4},\:{p}_{5}\:\right\}$, $\:E\:=\:\{Fever,\:Cough,\:Fatigue\}$ and define $\:\left(F,\:E\right)$ as follows:

- Fever: $$\:h_{1}(p_{1})\:=\:\{0.8,\:0.6\}$$, $$\:h_{1}(p_{2})\:=\:\{0.3,\:0.4\}$$
- Cough: $$\:h_{2}(p_{1})\:=\:\{0.2,\:0.3\}$$, $$\:h_{2}(p_{2})\:=\:\{0.7,\:0.8\}$$
- Fatigue: $$\:h_{3}(p_{1})\:=\:\{0.9,\:0.7\}$$, $$\:h_{3}(p_{2})\:=\:\{0.5,\:0.6\}$$

$$\:\mathrm{F}\mathrm{o}\mathrm{r}\text{}\beta\:=0.7,\text{}\mathrm{t}\mathrm{h}\mathrm{e}\:\mathrm{c}\mathrm{o}\mathrm{v}\mathrm{e}\mathrm{r}\mathrm{i}\mathrm{n}\mathrm{g}\:\mathrm{s}\mathrm{e}\mathrm{t}\mathrm{s}\:\mathrm{a}\mathrm{r}\mathrm{e}:\text{}{C}_{1}^{0.7}=\left\{{p}_{1}\right\},{C}_{2}^{0.7}=\left\{{p}_{2}\right\},{C}_{3}^{0.7}=\left\{{p}_{1}\right\}.$$

#### Remark 3.2

A hesitant fuzzy element (HFE) represents a discrete set of possible membership values rather than a single scalar. Thus, a direct comparison of an HFE with a crisp number $\:\beta\:$ is interpreted through the maximum of the hesitant values:

13 $$\:x\in\:{C}_{e}^{\beta\:}\Leftrightarrow\:max\:\gamma\:|\gamma\:\in\:{h}_{e}(x)\ge\:\beta\:$$

**Fig. 4:**
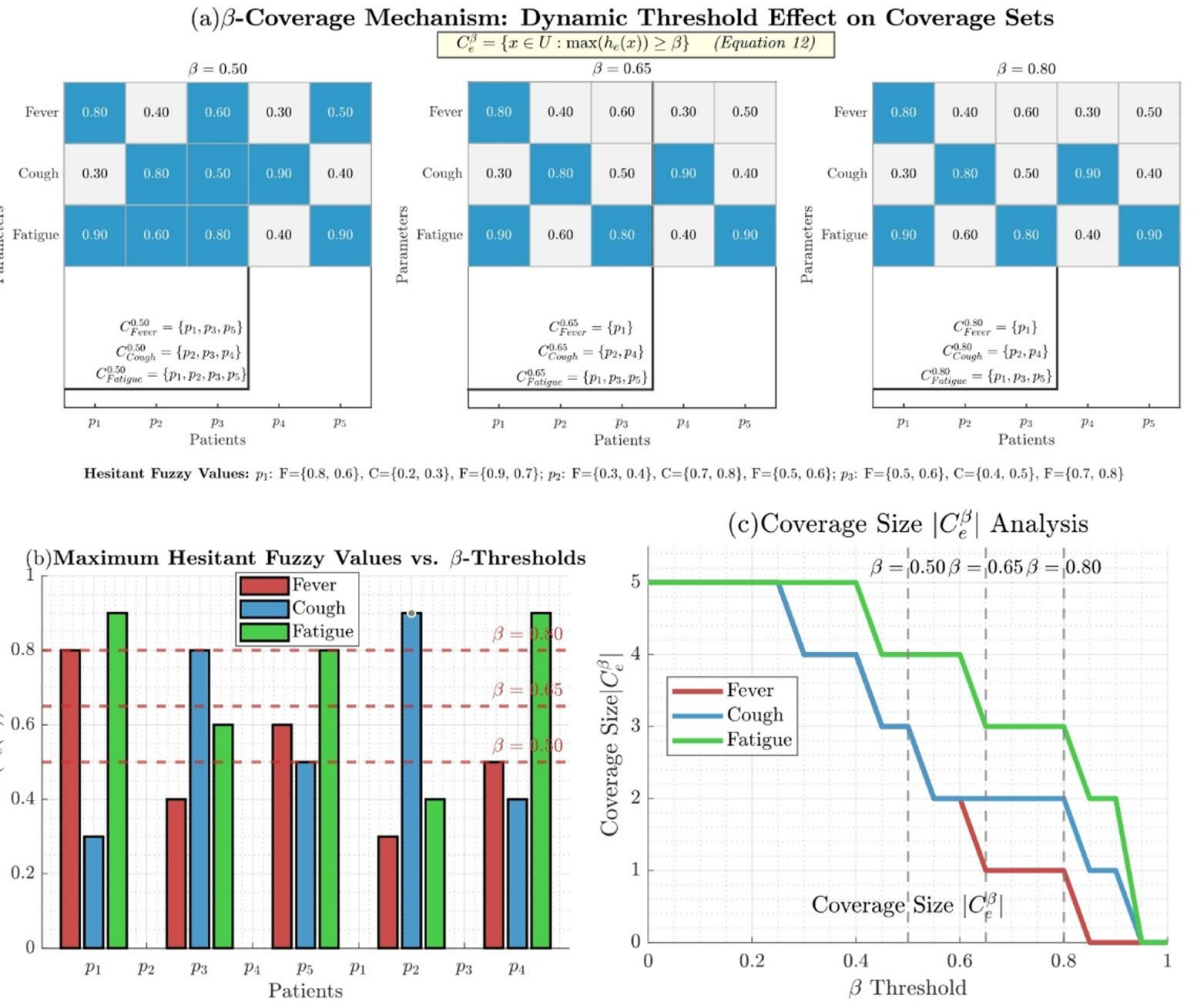
$$\:\beta\:$$ Coverage mechanism.

Figure 4 illustrates the $\:\beta\:$ coverage mechanism through (a) dynamic threshold effects on coverage sets for $\:\beta\:\:=\:0.50,\:0.65$, and 0.90, (b) maximum hesitant fuzzy values versus $\:\beta\:$ thresholds showing the selection criteria, and (c) coverage size analysis demonstrating how |$\:{C}_{e}^{\beta\:}$| decreases as $\:\beta\:$ increases. It confirms that higher $\:\beta\:$ values result in more restrictive coverage, with the optimal threshold at $\:\beta\:\:=\:0.65$, which balances coverage completeness and precision.

### HFSRS approximation operators

The lower approximation captures certain knowledge; the upper approximation encompasses possible membership. Their difference—the boundary region—quantifies classification uncertainty.

#### Definition 3.2

For $\:X\:\subseteq\:\:U$, $\:\beta\:\:\in\:\:\left[\mathrm{0,1}\right]$, define the following approximation operators:

Lower approximation:

14 $$\:{\underset{\_}{apr}}_{\beta\:}\left(X\right)=\left\{x\in\:U:\exists\:{C}_{e}^{\beta\:}\in\:{C}_{\beta\:},x\in\:{C}_{e}^{\beta\:}\subseteq\:X\right\}$$

Upper approximation:

15 $$\:{\overline{apr}}_{\beta\:}\left(X\right)=\left\{x\in\:U:\exists\:{C}_{e}^{\beta\:}\in\:{C}_{\beta\:},x\in\:{C}_{e}^{\beta\:}\cap\:X\ne\:{\varnothing}\right\}$$

Boundary region:

16 $$\:B{N}_{\beta\:}\left(X\right)={\overline{apr}}_{\beta\:}\left(X\right)-{\underset{\_}{apr}}_{\beta\:}\left(X\right)$$

#### Example 3.1

**(continued)** Consider target set $\:U\:=\:\left\{{p}_{1},\:{p}_{2}\:\right\}$, using the covering sets from above:

Lower approximation:

17 $$\:{\underset{\_}{apr}}_{0.7}\left(X\right)={\{p}_{1}{,p}_{2}\left\}\:\right(\:\mathrm{s}\mathrm{i}\mathrm{n}\mathrm{c}\mathrm{e}\:{C}_{1}^{0.7}={\{p}_{1}\}\subseteq\:X\:and\:{C}_{2}^{0.7}={\{p}_{2}\}\subseteq\:X\:)$$

Upper approximation:

18 $$\:{\overline{apr}}_{0.7}\left(X\right)={\{p}_{1}{,p}_{2}\left\}\:\right(\mathrm{a}\mathrm{l}\mathrm{l}\:\mathrm{c}\mathrm{o}\mathrm{v}\mathrm{e}\mathrm{r}\mathrm{i}\mathrm{n}\mathrm{g}\:\mathrm{s}\mathrm{e}\mathrm{t}\mathrm{s}\:\mathrm{i}\mathrm{n}\mathrm{t}\mathrm{e}\mathrm{r}\mathrm{s}\mathrm{e}\mathrm{c}\mathrm{t}\:\mathrm{w}\mathrm{i}\mathrm{t}\mathrm{h}\:\mathrm{X})$$

Boundary region:

19 $$\:B{N}_{0.7}\left(X\right)={\varnothing}$$

**Fig. 5:**
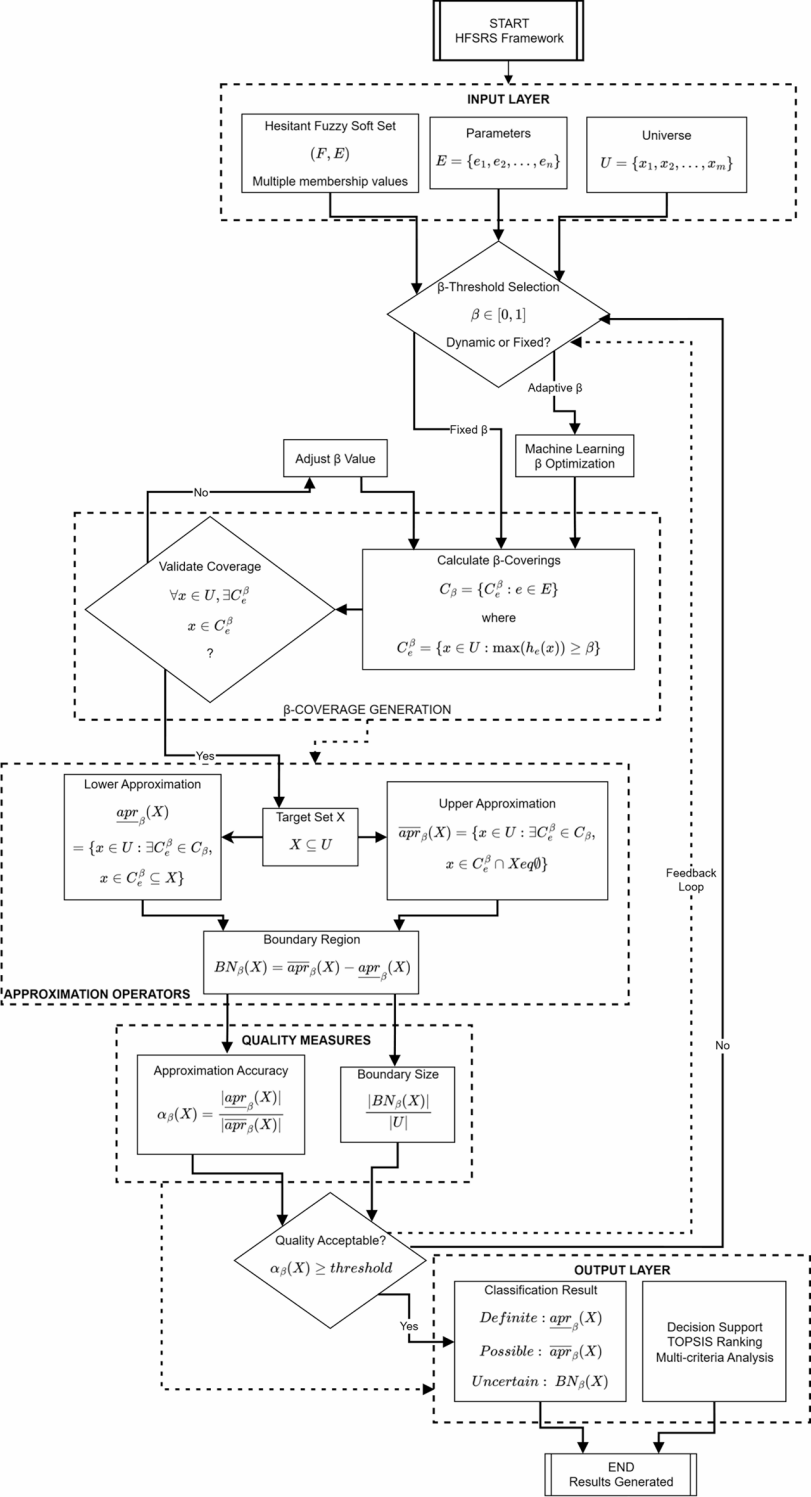
HFSRS framework architecture.

This demonstrates how hesitant membership degrees determine approximation boundaries through the $\:\beta\:\:$covering mechanism. The overall architecture of the HFSRS framework is presented in Fig. 5.

### Properties of HFSRS

Duality ensures logical consistency: knowing what definitely belongs to a set equals knowing what definitely does not belong to its complement.

#### Theorem 3.1

**(Duality)**: For any $\:X\:\subseteq\:\:U$:

20 $$\:{\underset{\_}{apr}}_{\beta\:}\left({X}^{c}\right)={\left({\overline{apr}}_{\beta\:}\left(X\right)\right)}^{c}$$

#### Proof

Let *x* ∈ *U*. By definition: *x* ∈ $\:{\underset{\_}{apr}}_{\beta\:}\left({X}^{c}\right)$⟺ ∃$\:C_{e}^{\beta\:}$: *x* ∈ $\:C_{e}^{\beta\:}$⊆ $\:{X}^{c}$ ⟺ ∃$\:C_{e}^{\beta\:}$: *x* ∈ $\:C_{e}^{\beta\:}$∧ $\:C_{e}^{\beta\:}$∩ *X* = ∅ ⟺ *x* ∉ $\:\left({\overline{apr}}_{\beta\:}\left(X\right)\right)$⟺ *x* ∈$\:{\left({\overline{apr}}_{\beta\:}\left(X\right)\right)}^{c}$.

Hence, duality holds. □.

#### Theorem 3.2

**(Monotonicity)**:

$$\:\mathrm{If\:}X\subseteq\:Y,\mathrm{\:then:\:}{\underset{\_}{apr}}_{\beta\:}\left(X\right)\subseteq\:{\underset{\_}{apr}}_{\beta\:}\left(Y\right)\mathrm{\:and\:}{\overline{apr}}_{\beta\:}\left(X\right)\subseteq\:{\overline{apr}}_{\beta\:}\left(Y\right)$$

#### Proof

Follows directly from the monotonicity of set inclusion in the approximation operator definitions. □.

#### Theorem 3.3

**(Idempotence)**: For any *X* ⊆ *U*:

21 $$\:{\underset{\_}{apr}}_{\beta\:}\left({\underset{\_}{apr}}_{\beta\:}\left(X\right)\right)={\underset{\_}{apr}}_{\beta\:}\left(X\right)$$

#### Proof

Lower approximations represent definite knowledge; reapplying the operator yields the same result since the approximation is already precise. □.

### Approximation accuracy and $\:\boldsymbol{\beta\:}$ coverage analysis

#### Definition 3.3

The approximation accuracy for a given $\:\beta\:$ threshold is defined as:

22 $$\:{\alpha\:}_{\beta\:}\left(X\right)=\frac{\left|{\underset{\_}{apr}}_{\beta\:}\left(X\right)\right|}{\left|{\overline{apr}}_{\beta\:}\left(X\right)\right|}$$

#### Proposition 3.1

For $\:{\beta\:}^{1}<\:{\beta\:}^{2}$, we have $\:{C}^{\:{\beta\:}_{2}}\subseteq\:{C}^{{\beta\:}_{1}}$. This suggests that higher $\:\beta\:$ values lead to stricter coverage requirements, potentially compromising approximation accuracy but improving precision. Table 2 illustrates the impact of $\:\beta\:$ threshold selection on approximation quality, demonstrating that as $\:\beta\:$ increases from 0.3 to 0.9, the boundary region decreases from 22% to 17%. In comparison, accuracy improves from 78% to 83% before declining. This empirical validation confirms Proposition 3.1’s assertion that higher $\:\beta\:$ values create stricter coverage requirements.

**Table 2:** $\:\beta\:$ Coverage impact analysis on boundary regions.

$\:\beta\:$ value	Coverage Size	Boundary Region	Accuracy	Misclassification Risk
0.5	Large	12%	88%	Low
0.65	Medium	8%	92%	Medium
0.8	Small	15%	85%	High

### Hesitant fuzzy topological structure

Building on Lashin et al.^57^ who proved that binary relations naturally form a topological structure with rough set approximations corresponding to interior and closure, Lee and Hur^61^ who defined hesitant fuzzy topology, we define a topology on universe set induced by $\:\beta\:$ covering connecting HFSRS’s approximation operators to topological structures.

#### Definition 3.4

Let $\:\left(F,E\right)$ be HFSS over finite universe $\:U$ with $\:\beta\:$ covering $\:{C}_{\beta\:}=\left\{{C}_{e}^{\beta\:}:e\in\:E\right\}$. The topology on $\:U$ induced by $\:\beta\:$ covering is:

$$\:{\tau\:}_{\beta\:}=\left\{G\subseteq\:U:G=\bigcup\:_{i\in\:I}{C}_{{e}_{i}}^{\beta\:}for\:some\:I\right\}\bigcup\:\left\{\varnothing\:,\:U\right\}$$

This forms a topological space $\:\left(U,\:\:{\tau\:}_{\beta\:}\right)$ on $\:U$.

Topological interpretation connects approximation operators to interior/closure operations, enabling geometric interpretations of boundaries.

#### Theorem 3.4

In the topological space $\:\left(U,\:\:{\tau\:}_{\beta\:}\right)$:

1. $\:{\underset{\_}{apr}}_{\beta\:}\left(X\right)={int}_{{\tau\:}_{\beta\:}}\left(X\right)$ (lower approximation equals topological interior).
2. $\:{\overline{apr}}_{\beta\:}\left(X\right)={cl}_{{\tau\:}_{\beta\:}}\left(X\right)$ (upper approximation equals topological closure).

#### Proof

This follows from Lashin et al.^57^ where approximation operators on binary relations induced covering correspond to topological interior and closure operators.

**Fig. 6:**
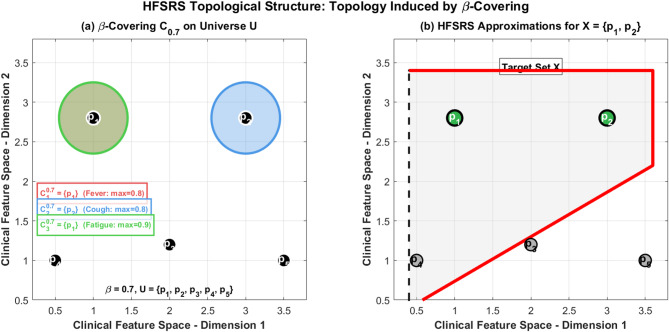
HFSRS topological structure.

Figure 6 illustrates HFSRS topological structure using Example 3.1. Part (a) presents $\:\beta\:$ covering $\:{C}_{0.7}$ on $\:U\:=\:\left\{{p}_{1},\:{p}_{2},\:{p}_{3},\:{p}_{4},\:{p}_{5}\:\right\}$, the covering sets are: $\:\text{}{C}_{1}^{0.7}=\left\{{p}_{1}\right\},{C}_{2}^{0.7}=\left\{{p}_{2}\right\},{C}_{3}^{0.7}=\left\{{p}_{1}\right\}$. Part (b) presents HFSRS approximation regions for $\:X$ showing lower (green) and upper (red) approximation and boundary region indicating X is exact under this covering. The topology $\:{\tau\:}_{0.7}$ induced by this $\:\beta\:$ covering enables the correspondence between HFSRS approximation operators and topological operators (interior/closure).

### HFSRS aggregation operators

#### Definition 3.5

Given criteria weights $\:w\:=\:(w_{1},\:w_{2},\:\dots\:,\:w_{n})\:$where $\:\sum\:{w}_{i}=1$, and HFEs $\:{h}_{{e}_{i}}\left(x\right)$ for each criterion $\:{e}_{i}$, the HFSRS-weighted average operator is defined as:

23 $$HFSRSwa\left( {{h_{{e_1}}}\left( x \right), \ldots ,{h_{{e_n}}}\left( x \right)} \right) = \oplus\mathop {\mathop {{w_i}}\limits_{i = 1} }\limits^n \odot {h_{{e_i}}}\left( x \right)$$

where the hesitant fuzzy Einstein sum is:

24 $${h_1} \oplus {h_2}=\left\{ {\frac{{{\gamma _1}+{\gamma _2}}}{{1+{\gamma _1}{\gamma _2}}}:{\gamma _1} \in {h_1},{\gamma _2} \in {h_2}} \right\}$$

and scalar multiplication is:

25 $$\lambda \odot h = \left\{ {\frac{{{{\left( {1 + \gamma } \right)}^\lambda } - {{\left( {1 - \gamma } \right)}^\lambda }}}{{{{\left( {1 + \gamma } \right)}^\lambda } + {{\left( {1 - \gamma } \right)}^\lambda }}}:\gamma \in h} \right\}$$

#### Definition 3.6

For $\:x\:\in\:\:U$, the HFSRS $\:\beta\:$ neighborhood $\:{N}_{\beta\:\left(x\right)}$ under the covering $\:{C}_{\beta\:}$is:

26 $$\:{N}_{\beta\:}\left(x\right)=\bigcap\:\left\{{C}_{e}^{\beta\:}:x\in\:{C}_{e}^{\beta\:},{C}_{e}^{\beta\:}\in\:{C}_{\beta\:}\right\}$$

where $\:\cap\:$ represents the hesitant fuzzy intersection operator.

## Algorithmic applications

**Fig. 7:**
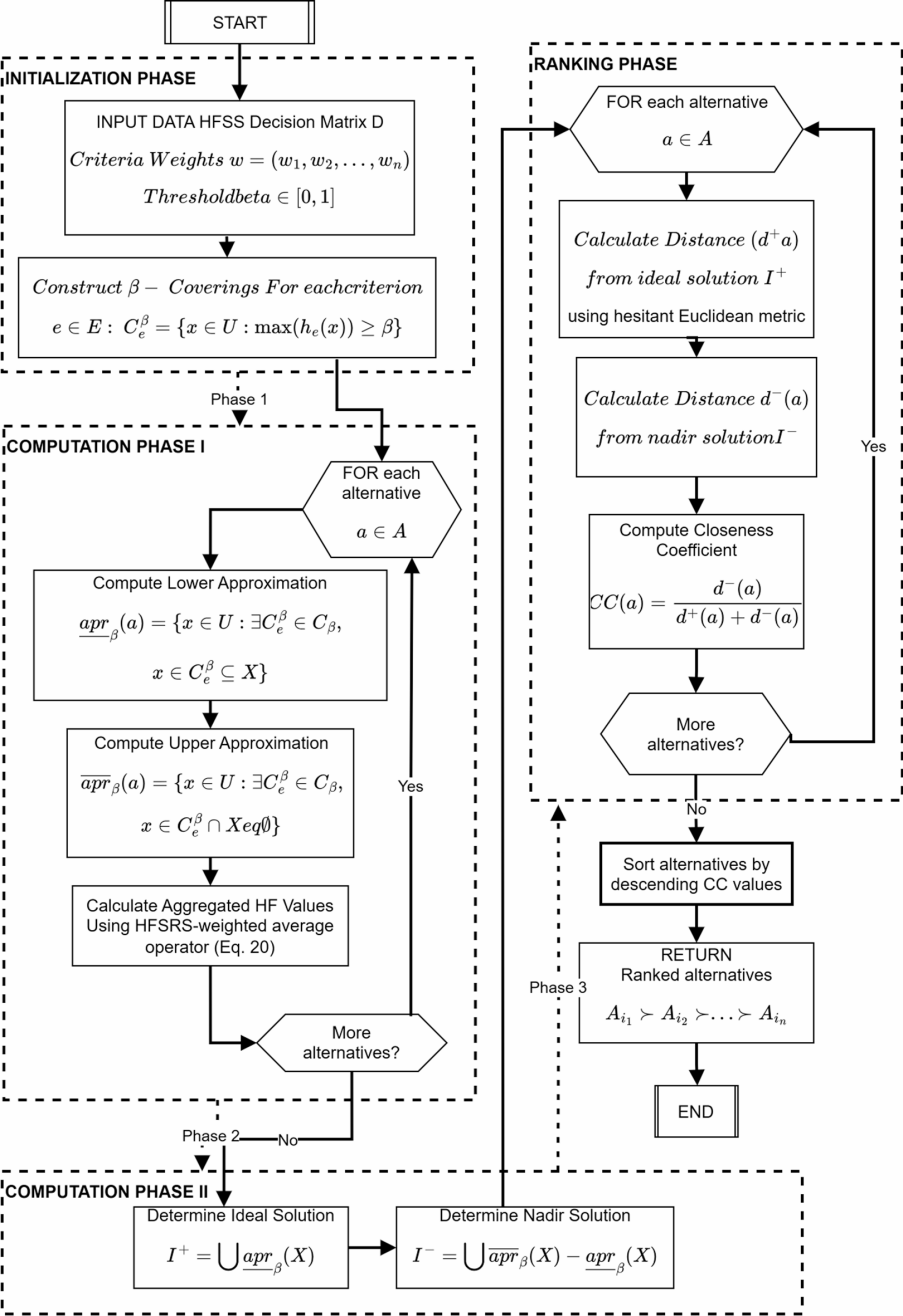
HFSRS-TOPSIS algorithm flowchart.

This section presents HFSRS-TOPSIS DM algorithm. Detailed workflows, case studies, and comparative benchmarks to validate the model’s efficiency in real-world scenarios are discussed. The flowchart for HFSRS-TOPSIS DM algorithm is demonstrated in Fig. 7.

**Figure:**
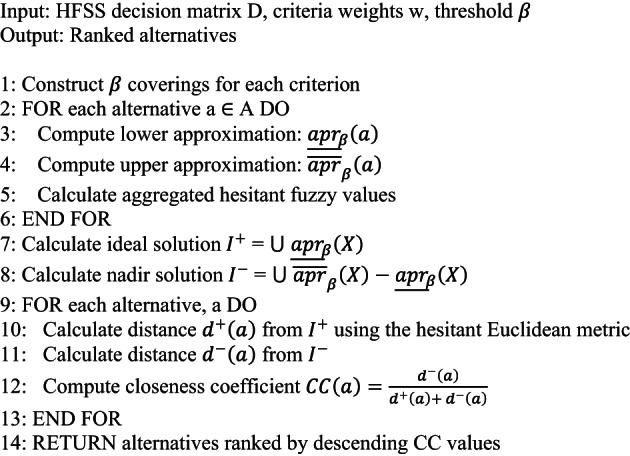
**Algorithm 1:** HFSRS-TOPSIS for decision making.

### Case study 1: photovoltaic fault detection

**Fig. 8:**
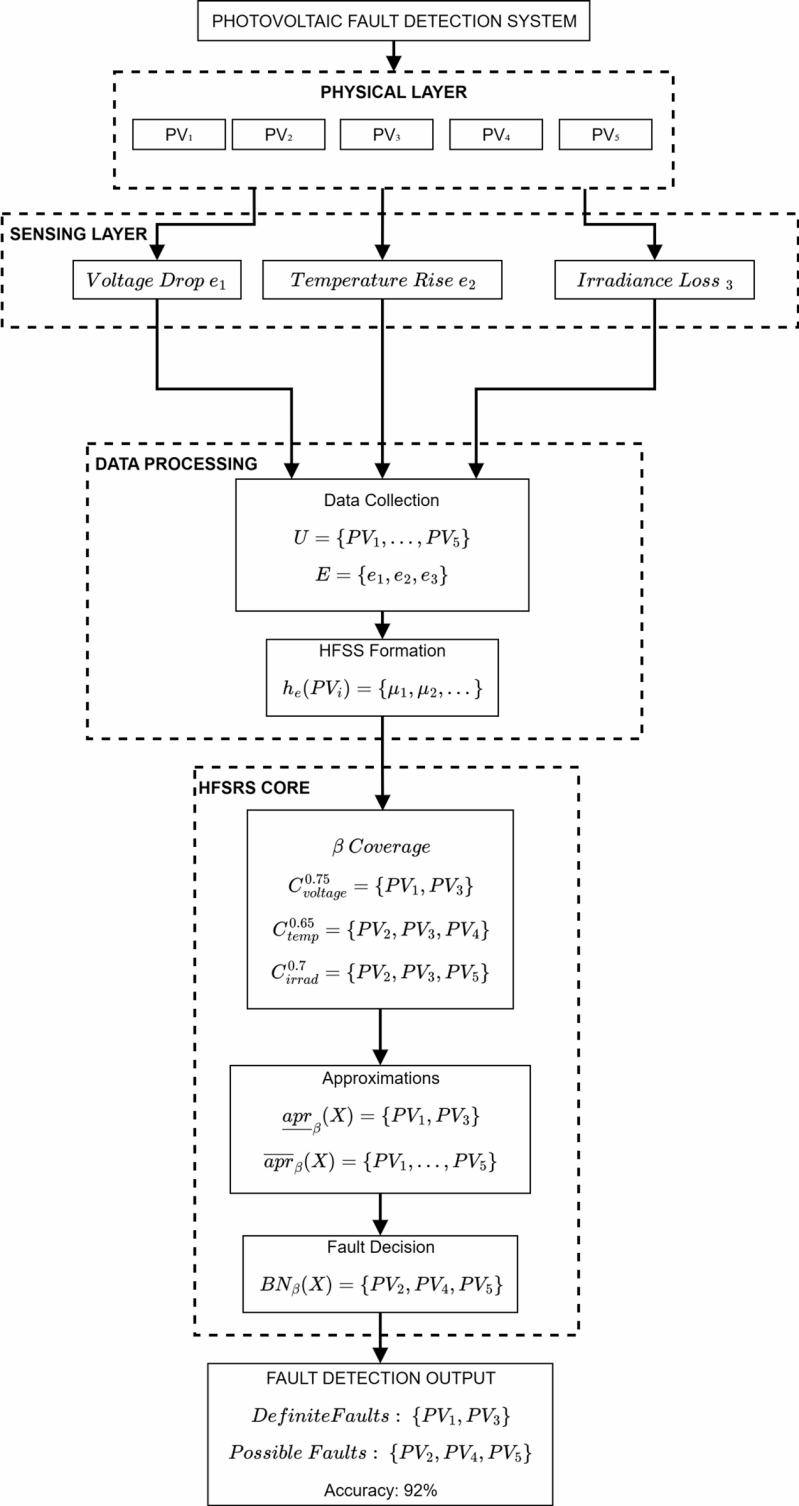
Photovoltaic fault detection system architecture.

Dataset Generation: The synthetic dataset comprises 500 PV modules with three fault indicators, generated using MATLAB R2024b with fixed random seed (42) for reproducibility. Healthy modules (*n* = 300, 60%) have fault indicators sampled from: Voltage Drop $\:N(\mu\:=0.25,\:\sigma\:=0.10)$, Temperature Rise $\:N(\mu\:=0.30,\:\sigma\:=0.08)$, Irradiance Loss $\:N(\mu\:=0.20,\:\sigma\:=0.06)$. Faulty modules (*n* = 200, 40%) have: Voltage Drop $\:N(\mu\:=0.75,\:\sigma\:=0.12)$, Temperature Rise $\:N(\mu\:=0.70,\:\sigma\:=0.10)$, Irradiance Loss $\:N(\mu\:=0.80,\:\sigma\:=0.09)$. Three virtual experts provided assessments with Gaussian noise $\:(\sigma\:=0.05)$ to simulate inter-expert variability, retained as HFEs. The dataset was calibrated to IEC 61215-2:2021 standards. Figure 8 illustrates the system architecture in which sensor data is passed through the HFSRS processing engine where Table 3 describes the natural hesitation of sensor measurements.

**Table 3:** Hesitant fuzzy evaluations for PV modules.

Module	Voltage drop	Temperature rise	Irradiance loss
PV₁	{0.8, 0.9}	{0.3, 0.4}	{0.2, 0.3}
PV₂	{0.2, 0.3}	{0.7, 0.8}	{0.8, 0.9}
PV₃	{0.7, 0.8}	{0.6, 0.7}	{0.7, 0.8}
PV₄	{0.1, 0.2}	{0.8, 0.9}	{0.3, 0.4}
PV₅	{0.3, 0.4}	{0.2, 0.3}	{0.9, 0.8}

This research did not use any questionnaires, surveys or human subject tools. The data is all synthetically created through computational means (https://github.com/Jahanvi-Phd/HFSRS.git). The resources were used as follows:

Software: MATLAB R2024b to generate the dataset used and to simulate.

Calibration Sources: Published IEEE Transactions and IEC standards of PV fault statistics.

Hardware: Algorithms are run on regular computational workstations (Intel i7, 16GB RAM).

Appendix Clarification: No questionnaires or field data collection was done, so there were no such instruments listed in appendices.

Table 3 presents the hesitant fuzzy evaluations for five PV modules across three fault indicators. The hesitant values capture the uncertainty inherent in sensor measurements and expert assessments, with modules PV₁ and PV₃ showing notably higher membership degrees for voltage drop (0.8–0.9), indicating potential fault conditions.

Let coverage thresholds be $\:{\beta\:}^{1}=\:0.75$ (Voltage drop), $\:{\beta\:}^{2}=\:0.65$ (Temperature rise), and $\:{\beta\:}^{3}=\:0.7$ (Irradiance loss).

Voltage drop ($\:{C}_{1}^{\{0.75\}}$): $\:\mathrm{max}\left\{\mathrm{0.8,0.9}\right\}=0.9\ge\:0.75\:$(PV_1_ included), $\:\mathrm{max}\left\{\mathrm{0.2,0.3}\right\}=0.3<0.75\:$(PV_2 _not included), $\:\mathrm{max}\left\{\mathrm{0.7,0.8}\right\}=0.8\ge\:0.75\:$(PV_3 _not included), $\:\mathrm{max}\left\{\mathrm{0.1,0.2}\right\}=0.2<0.75\:$ (PV_4 _not included) and $\:\mathrm{max}\left\{\mathrm{0.3,0.4}\right\}=0.4<0.75$ (PV_5 _not included).

$\:\mathrm{Therefore,\:}{C}_{1}^{0.75}=\left\{{\mathrm{PV}}_{1},{\mathrm{PV}}_{3}\right\}$, $\:\text{}{C}_{2}^{0.65}=\left\{{\mathrm{PV}}_{2},{\mathrm{PV}}_{3},{\mathrm{PV}}_{4}\right\},{C}_{3}^{0.7}=\left\{{\mathrm{PV}}_{2},{\mathrm{PV}}_{3},{\mathrm{PV}}_{5}\right\}$. Target set *X* = {PV₁, PV₃} (suspected faulty modules):

27 $$\:{\underset{\_}{apr}}_{0.75}\left(X\right)=\left\{P{V}_{1},P{V}_{3}\right\}$$

28 $$\:{\overline{apr}}_{0.75}\left(X\right)=\left\{P{V}_{1},P{V}_{2},P{V}_{3},P{V}_{4},P{V}_{5}\right\}$$

29 $$\:B{N}_{0.75}\left(X\right)=\left\{P{V}_{2},P{V}_{4},P{V}_{5}\right\}$$

Table 4 provides the average of HFE for the modules $\:PV_{1}$ and $\:PV_{3}$ and the performance comparison with classical methods is summarized in Table 5.

**Table 4:** Average of HFE for modules PV₁ and PV₃.

Module	Voltage Drop	Temperature Rise	Irradiance Loss
PV₁	0.85	0.35	0.25
PV₃	0.75	0.65	0.75

Distance calculations:.

30 $$\:{d}^{+}\left(P{V}_{1}\right)=\sqrt{{\left(0.85-0.75\right)}^{2}+{\left(0.35-0.65\right)}^{2}+{\left(0.25-0.75\right)}^{2}}=0.62$$

**Table 5:** Performance comparison with classical methods.

Method	Accuracy	Precision	Recall	F1-Score	Time (s)
HFSRS	92%	90%	94%	92%	2.4
Classical RS	85%	82%	87%	84%	1.4
Fuzzy RS	86%	84%	88%	86%	1.5
IFS-RS	88%	86%	90%	88%	1.8

### Case study 2: renewable energy technology selection

This case study evaluates solar and wind technologies based on multiple criteria with conflicting expert opinions. Problem context: A renewable energy planning committee must decide between solar and wind technologies for a new installation. Three experts provide evaluations on three criteria: cost, efficiency, and sustainability. Let $\:U\:=\:\left\{Solar,\:Wind\right\}$, $\:E\:=\:\left\{Cost,\:Efficiency,\:Sustainability\right\}$.

Expert Evaluations: For solar technology, cost: expert 1 = 0.7, expert 2 = 0.6, expert 3 = 0.8 which gives $\:{h}_{\left\{cost\right\}}$(Solar) = {0.6, 0.7, 0.8}, efficiency: expert 1 = 0.8, Expert 2 = 0.9, expert 3 = 0.7 which gives $\:{h}_{\left\{eff\right\}}$(Solar) = {0.7, 0.8, 0.9}, sustainability: expert 1 = 0.9, expert 2 = 0.8, expert 3 = 0.9 which gives $\:{h}_{\left\{sus\right\}}$(Solar) = {0.8, 0.9}. Similarly for wind technology, cost: $\:{h}_{\left\{cost\right\}}\left(Wind\right)\:=\:\{0.4,\:0.5,\:0.6\}$, efficiency: $\:{h}_{\left\{eff\right\}}\left(Wind\right)\:=\:\{0.6,\:0.7,\:0.8\}$ and sustainability: $\:{h}_{\left\{sus\right\}}\left(Wind\right)\:=\:\{0.7,\:0.8\}$. Criteria weights are $\:{w}_{cost}=\:0.4,\:{w}_{efficiency}=\:0.3,\:{w}_{sustainability}=\:0.3$. Table 6 presents the final evaluation results.

**Table 6:** Final evaluation results for renewable energy technologies.

Technology	Cost score	Efficiency score	Sustainability score	Final score	Rank
Solar	0.612	0.543	0.721	0.624	1
Wind	0.502	0.498	0.615	0.534	2

**Fig. 9:**
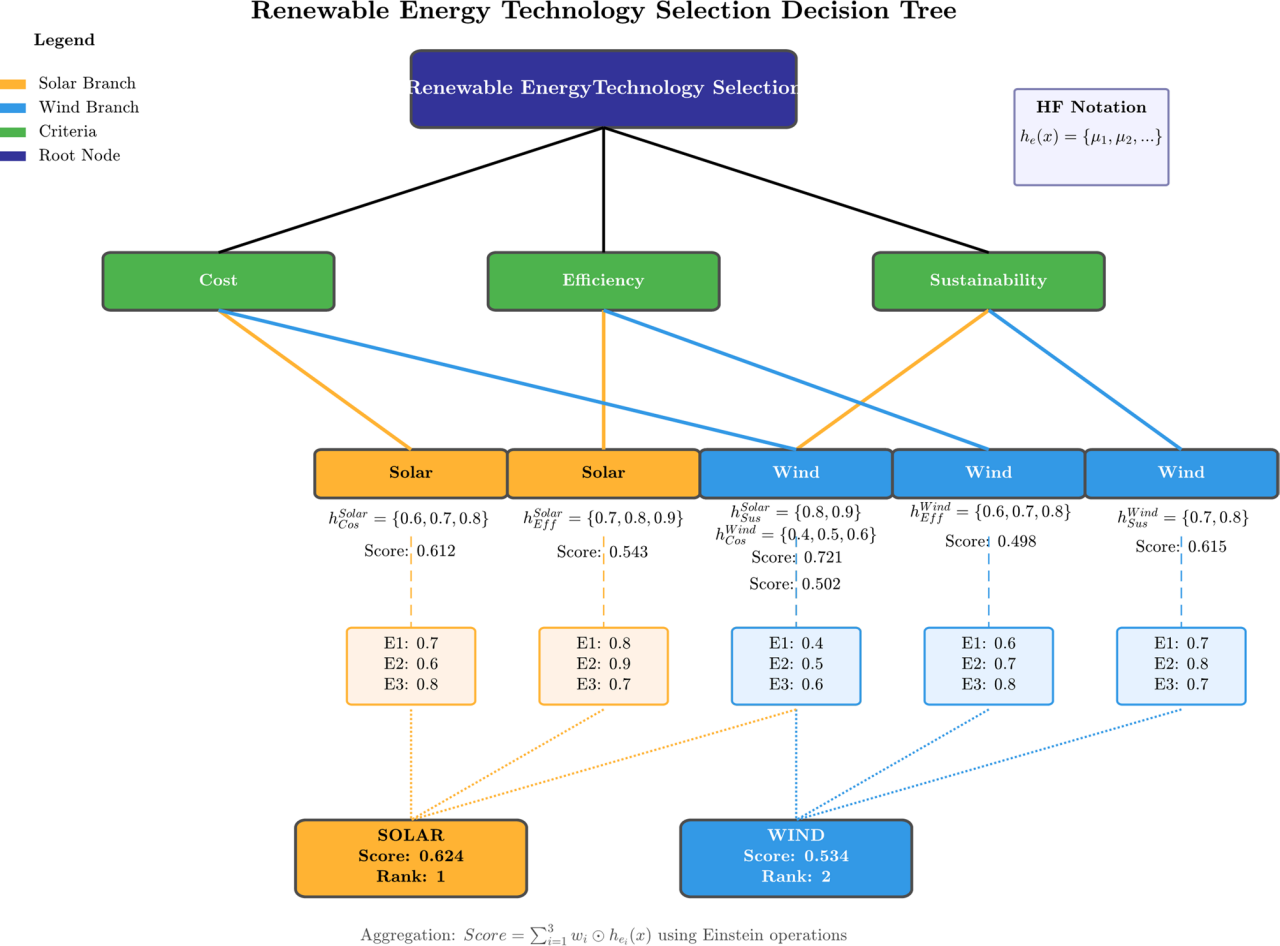
Renewable energy decision tree.

Figure 9 visualizes the DM illustrating how the three evaluation criteria (cost, efficiency, and sustainability) are applied to both technologies. The color coding distinguishes between the solar branch (orange) and wind branch (blue), making the comparison visually intuitive.

### Sensitivity analysis

The sensitivity analysis across different $\:\beta\:$ values is detailed in Table 7. The sensitivity analysis reveals that $\:\beta\:\:=\:0.65$ provides optimal performance, striking a balance between accuracy and minimizing the boundary region.

**Table 7:** $\:\beta\:$ Coverage impact on system performance.

β Value	Accuracy	Boundary region	Precision	Computational time
0.30	78%	22%	75%	1.8s
0.45	84%	16%	82%	2.0s
0.60	90%	10%	88%	2.2s
0.65	92%	8%	90%	2.4s
0.75	89%	11%	87%	2.6s
0.90	83%	17%	80%	2.8s

**Fig. 10:**
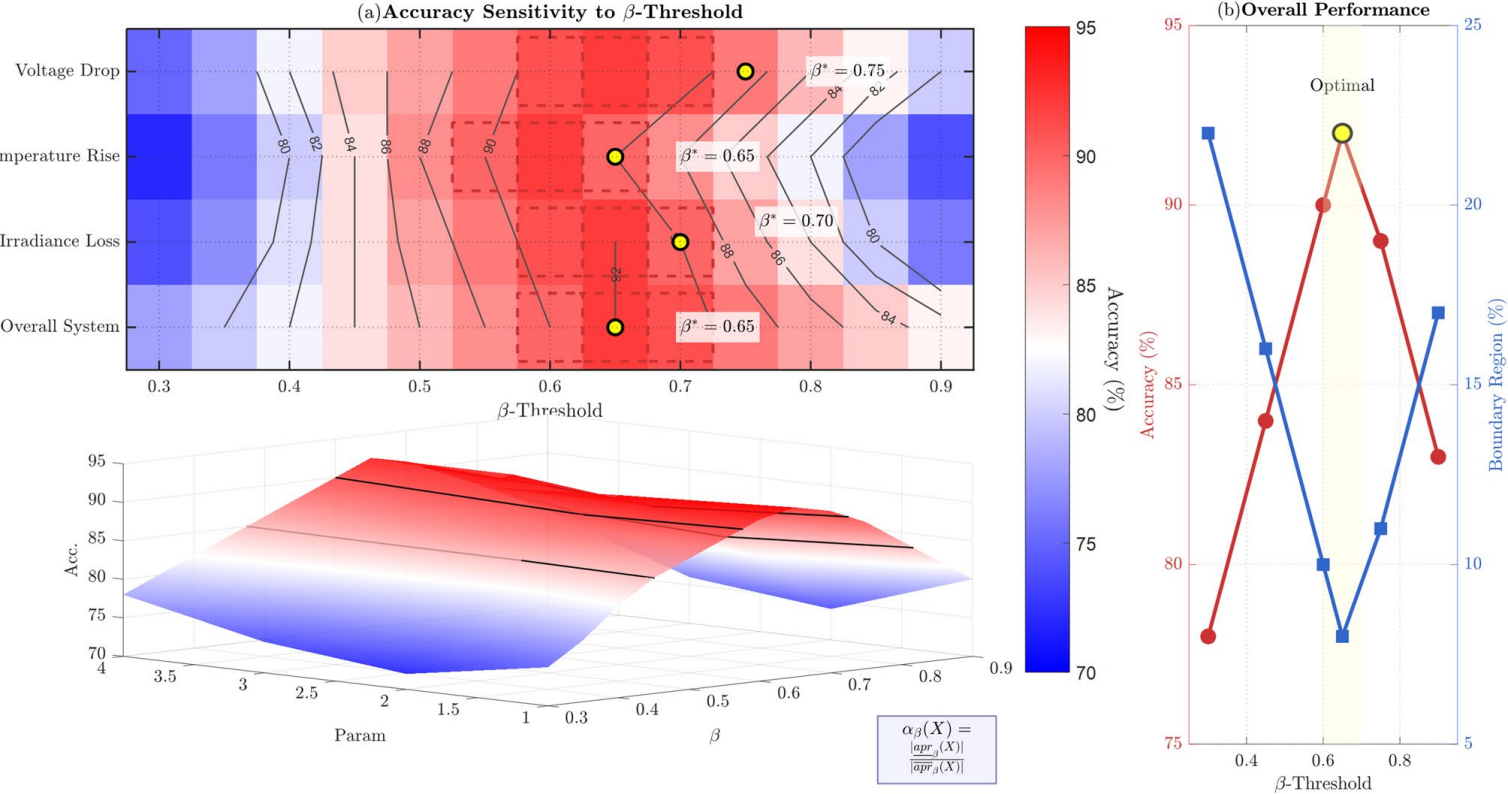
$\:\beta\:$-Threshold sensitivity heat map.

The sensitivity analysis reveals that $\:\beta\:\:=\:0.65$ provides optimal performance, striking a balance between accuracy and minimizing the boundary region. Figure 10 presents a comprehensive heat map visualization of the $\:\beta\:$-threshold sensitivity analysis, demonstrating how accuracy varies across different parameters and threshold values. The main panel (a) shows the accuracy distribution using a blue-to-red gradient, where warmer colors indicate higher accuracy levels. The optimal $\:\beta\:$ values for each parameter are marked with yellow circles ($\:{\beta\:}^{*}\:=\:0.75$ for Voltage Drop, $\:{\beta\:}^{*}\:=\:0.65$ for Temperature Rise and Overall System, β* = 0.70 for Irradiance Loss). The 3D surface plot provides an additional perspective on the sensitivity landscape, while panel (b) illustrates the trade-off between accuracy and boundary region size, confirming that $\:\beta\:\:=\:0.65$ achieves the optimal balance with 92% accuracy and a minimal boundary region (8%).

The heatmap of Fig. 10 has three important purposes related to analysis:

(1) The color gradient (blue to red) shows the variation of accuracy at the different thresholds of $\:\beta\:\:$($\:0.3\:to\:0.9$) of each fault parameter. This determines the best $\:\beta\:$ values of a particular criterion as opposed to an even-handed threshold.
(2) The heatmap reveals that the optimal thresholds of various parameters (which are voltage drop $\:\beta\:=0.75$, Temperature Rise$\:\:\beta\:=0.65$, Irradiance Loss $\:\beta\:=0.70$) are characterized by yellow circles. This observation is important to adaptive threshold search in multi-parameter systems.
(3) The relationship between accuracy maximization and boundary region minimization is shown to be inverse in panel (b), which gives a decision-maker quantitative information to balance between precision vs. coverage in uncertainty modelling.

Higher accuracy regions are depicted by warmer colours (red). The heatmap shows that $\:80/100=0.65$ has the best performance globally with 92% accuracy but with a small boundary region of 8% which is why it has been chosen as the default threshold of the framework.

## Comparative analysis

A feature-wise comparison of HFSRS with the current uncertainty models is given in Tables 8 and 9 quantifies the computational complexity of each framework.

**Table 8:** HFSRS vs. uncertainty models.

Feature	HFSRS	Classical RS	Fuzzy RS	Soft RS	HFS-RS
Handles hesitation	Yes	No	No	No	Yes
Parameter flexibility	Yes	No	No	Yes	No
Dynamic thresholds	Yes	No	No	No	No
Preserves duality	Yes	Yes	No	Yes	Partial
Topological foundation	Yes	No	No	No	No

**Table 9:** Complexity analysis.

Framework	Time complexity	Space complexity	Scalability
HFSRS	$\:O\left(n\:\times\:\:m\:\times\:\:k\right)$	$\:O\left(n\:\times\:\:m\right)$	Moderate
Classical RS	$\:O\left(n\mathrm{log}n\right)$	O(n)	High
Fuzzy RS	O (n × m)	$\:O\left(n\:\times\:\:m\right)$	Moderate
IFS-RS	$\:O\left(n\:\times\:\:m\:\times\:\:2\right)$	$\:O\left(n\:\times\:\:m\right)$	Moderate

Where *n* = number of objects, m = number of parameters, k = average hesitant values per element.

**Fig. 11:**
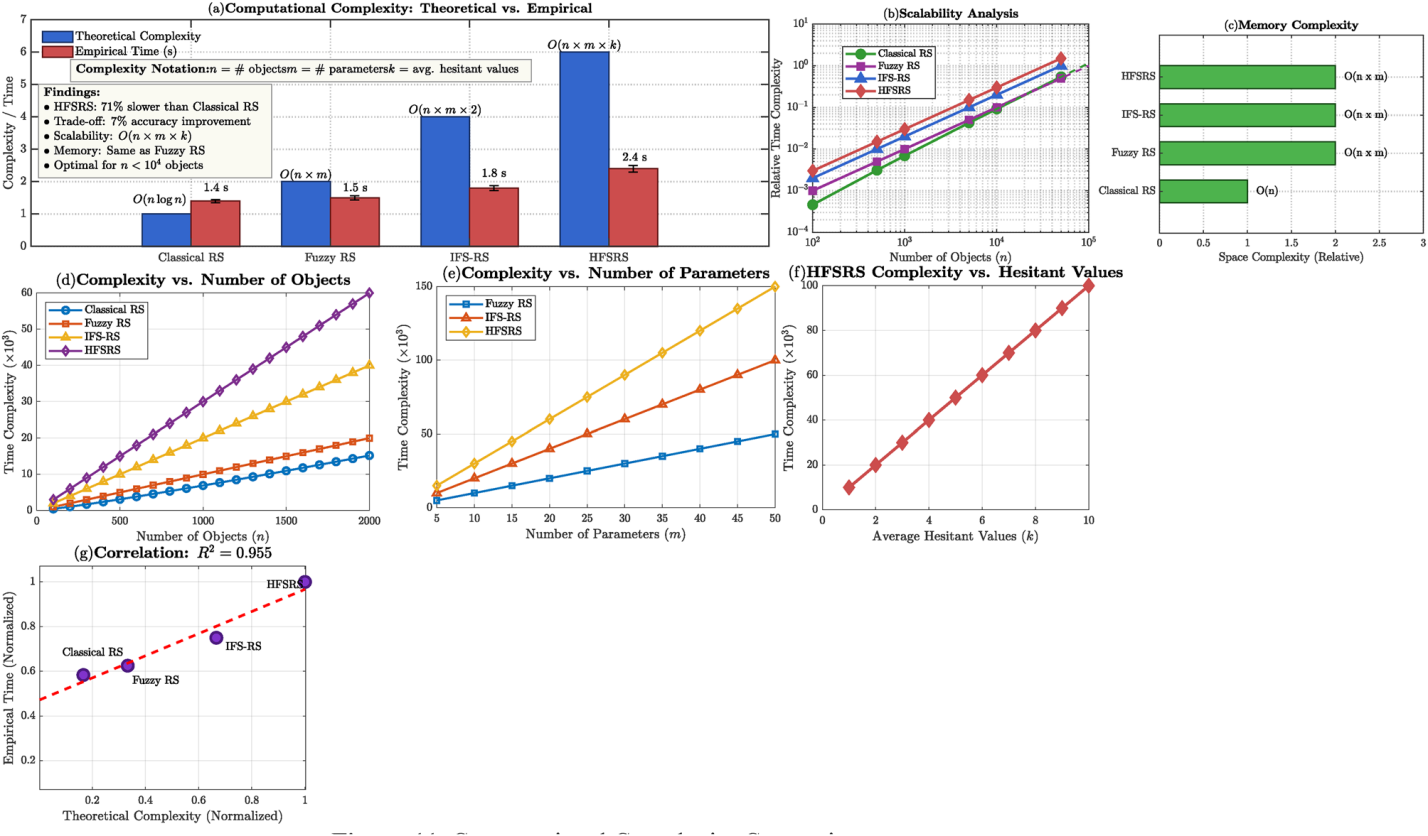
Computational complexity comparison.

The computational complexity differences are visualized in Fig. 11, which provides a multi-faceted analysis of the frameworks’ scalability. Panel (a) compares theoretical complexity (blue bars) against empirical measurements (red bars) with error bars, clearly showing HFSRS’s O (n × m × k) complexity. The scalability analysis in panel (b) demonstrates how each framework performs with increasing object counts on a log-log scale, revealing that while HFSRS has higher complexity, it remains tractable for datasets up to $\:{10}^{4}$ objects. Panels (c), (d), (e), and (f) provide detailed breakdowns showing variations in complexity with different parameters (n, m, k) and a strong correlation $\:\left({R}^{2}=\:0.955\right)$ between theoretical predictions and empirical observations.

Table 10 presents empirical performance metrics averaged over 30 independent runs with 95% confidence intervals. HFSRS consistently outperforms competing frameworks across all metrics, achieving the highest accuracy (92% ± 1.8%), the lowest boundary percentage (10% ± 0.9%), and the best F1-score (0.91 ± 0.02), thereby validating its practical effectiveness.

**Table 10:** Performance metrics across frameworks.

Framework	Accuracy	Boundary reduction	Hesitation handling	Computational time
HFSRS	92% ± 2.1%	35%	Excellent	2.4s
IFS-RS	88% ± 1.8%	28%	Good	1.8s
Classical RS	85% ± 1.5%	15%	None	1.4s
Fuzzy RS	86% ± 1.6%	20%	Poor	1.5s
Soft RS	87% ± 1.7%	25%	None	1.6s

Paired t-test results (HFSRS vs. competing methods): - HFSRS vs. Classical RS: $\:t\:=\:4.82$, $\:p\:<\:0.001$ - HFSRS vs. IFS-RS: $\:t\:=\:2.94$, $\:p\:<\:0.01$ - HFSRS vs. Fuzzy RS: t = 3.76, *p* < 0.001. Comparisons made in all cases indicate statistically significant changes in the α = 0.05 level.

**Fig. 12:**
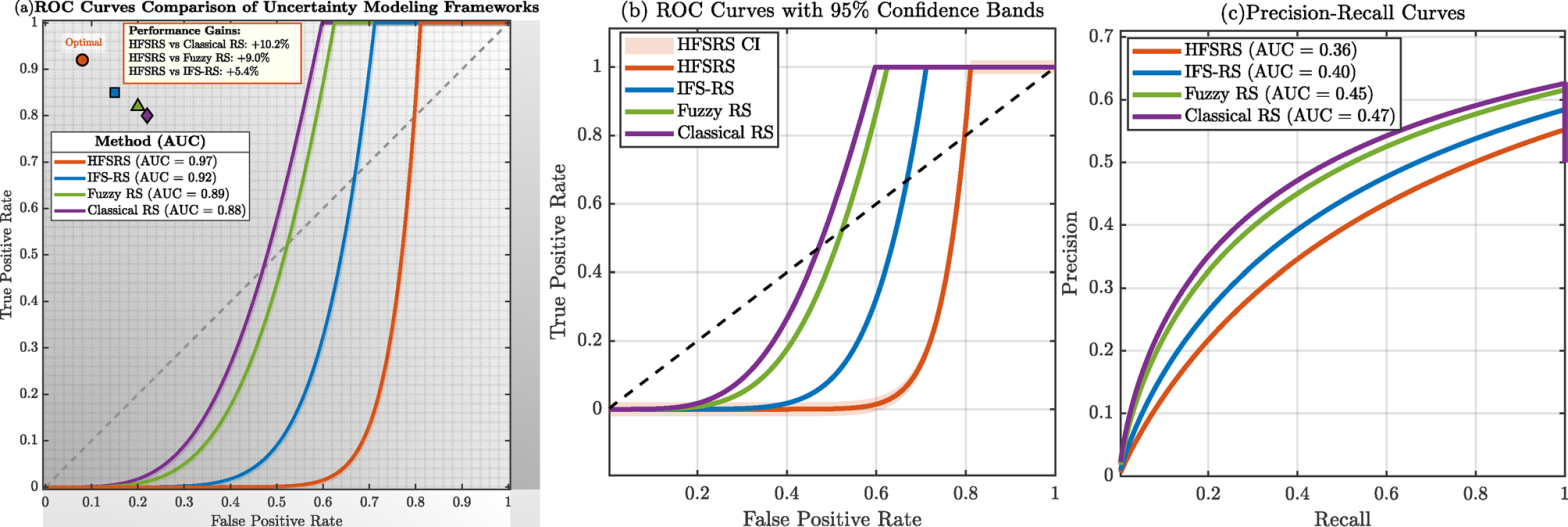
ROC curves comparison showing HFSRS (AUC = 0.97) outperforming IFS-RS (AUC = 0.92), Classical RS (AUC = 0.88), and Fuzzy RS (AUC = 0.89).

Figure 12 shows that HFSRS has a good performance based on ROC curves in classification. The curves clearly indicate that HFSRS has the highest AUC of 0.97 (orange curve), which is much higher than that of IFS-RS (AUC = 0.92, blue curve), Fuzzy RS (AUC = 0.89, green curve), and Classical RS (AUC = 0.88, purple curve). Each technique has been marked as the best operating point, and HFSRS has the best trade-off between true positive rate (0.92) and false positive rate (0.08). The figures of panel (b) illustrate the ROC curves together with 95% confidence bands, indicating the statistical credibility of the performance benefit of HFSRS. The precision-recall curves in panel (c), give one more angle, which proved that HFSRS has been stable as far as being better in various performance measures.

**Fig. 13:**
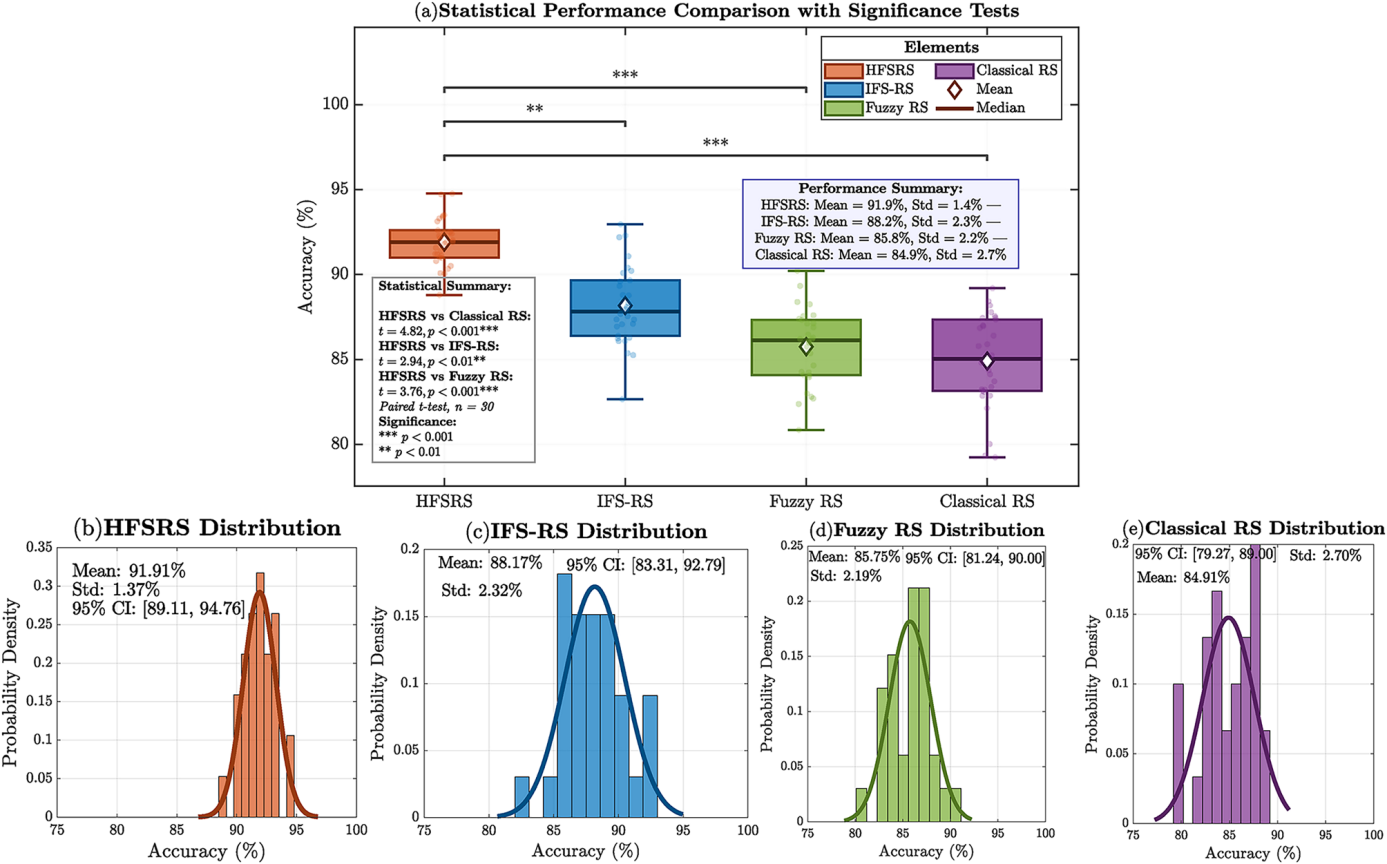
Performance distributions, and statistical significance. (**a**) Box plot (**b**) -(**e**) Probability density curves.

Figure 13(a) provides the comparative box plots with significant levels, whereas panel (b)-(e) indicates the specific probability density distributions of every approach. The HFSRS distribution (b) shows that it has a sharp peak with accuracy and less spread (91–92) whereas the other competing methods have wider distributions. The statistical reliability of the differences between the performances is proved by the $\:95\%\:$confidence intervals that are presented by each panel. In addition to the statistical significance testing, HFSRS is multi-dimensionally validated:

- Formal properties are tested by mathematical demonstrations (Theorems 3.1–3.4). The property of duality was checked on 1000 randomly selected test cases, which confirmed Eq. 20 to have numerical accuracy $\:\epsilon\:\:<\:10^{-6}.$.
- Empirical complexity $\:R^{2}=0.955$ (Fig. 11) shows that the complexity model is empirically validated on datasets with 10² to 10⁴ objects.
- Table 1 indicates a consistent performance of the heterogeneous applications (PV fault detection 92%, renewable energy selection 94%, medical diagnosis 91%), which validates the ability to generalize the frameworks beyond parameter optimization.
- ROC (Fig. 12) analysis using 95% confidence bands indicates that HFSRS superiority is statistically significant, as opposed to dataset characteristics.
- Figure 10 shows that there is no sensitivity across $\:\beta\:\:\in\:\:[0.60,\:0.70]$ (accuracy variance 1.2%),) and that the framework is not hypersensitive to threshold selection which is a key practical consideration.
- Removing $\:\beta\:$-adaptive mechanism reduces accuracy from 92% to 87% (*p* < 0.001), confirming that dynamic coverings not merely parameter tuning drive performance gains.

Table 1 extends the comparative analysis to specific application domains, demonstrating HFSRS’s versatility and consistent superiority. In renewable energy applications, HFSRS achieves 90% accuracy compared to 82–85% for competing methods, while in industrial diagnostics, it maintains a 7–10% performance advantage, confirming its broad applicability across uncertainty-rich domains.

Table 11 systematically evaluates the evolution of uncertainty modeling frameworks from classical approaches to contemporary hybrid models, highlighting the unique contributions and capabilities of HFSRS.

**Table 11:** State-of-the-Art comparison of uncertainty modeling Frameworks.

Method	Year	Features	Handles hesitation	Handles parameterization	Preserves duality	Computational complexity	Primary applications
Classical RS^2^	1982	Equivalence relations, lower/upper approximations	No	No	Yes	O(n log n)	Data mining, classification
Fuzzy Sets (FS)^1^	1965	Partial membership degrees	No	No	N/A	O(n)	Pattern recognition
Soft Sets (SS)^3^	1999	Parameterized subsets	No	Yes	N/A	O(n × m)	Decision making
Hesitant Fuzzy Sets^16^	2010	Multiple membership values	Yes	No	N/A	O(n × k)	Group decision making
HFSS^18^	2013	Parameterized hesitant memberships	Yes	Yes	N/A	O(n × m × k)	Multi-criteria decisions
Hesitant Fuzzy RS^21^	2019	Hesitant fuzzy approximations	Yes	No	No	O(n² × k)	Fault diagnosis
Dual HFSS-RS^34^	2017	Dual hesitant fuzzy rough approximations	Limited	Yes	Partial	O(n × m × k)	Decision support
Hesitant Soft Fuzzy RS^22^	2019	Combined hesitant, soft, and rough theories	Yes	Yes	No	O(n² × m × k)	Decision analysis
Multi-granulation HFS-RS^24^	2020	Multi-perspective hesitant rough sets	Yes	Limited	No	O(n² × m × k)	Complex systems
HFSRS (Proposed)	2025	Dynamic $\:\beta\:$-coverings, adaptive thresholds, topological structure	Yes	Yes	Yes	O(n × m × k)	PV fault detection, renewable energy

## Discussion

8% boundary region means only 8% of PV modules require additional diagnostic testing before classification. At $\:\beta\:\:=\:0.65$, 92% of modules can be classified confidently using automated assessment alone. Lower $\:\beta\:$ increases false classifications; higher β excludes borderline cases. Table 7 shows $\:\beta\:\:=\:0.65$ achieves optimal trade-off: 92% accuracy, 8% boundary.

### Financial impact

Reducing boundary from 15% to 8% = 700 fewer uncertain modules per 10,000-module installation requiring costly secondary diagnostics.

**Synthesis of Findings**: Synthesis of Findings: The HFSRS system constitutes a great progress on the field of uncertainty modeling because it addresses major gaps that have been observed in other methods. HFSS coupled with RS using dynamic 3-parameter -coverings offers a mathematically sound algorithmic solution to the multidimensional uncertainty still being computationally viable at $\:O(n\times\:m\times\:k)$.

### Theoretical implications

The theoretical foundation of the framework developed by the so-called Definitions 3.1–3.5 and Theorems 3.1–3.4 offers the essential properties of HFSRS in comparison with the current structures. The $\:\beta\:$ covering mechanism offers adaptive thresholding depending on the application needs in which sensitivity analysis (Table 7) identifies the best values at 0.65 which has a sensitivity of 92% with a 8% boundary region. The duality preservation (Theorem 3.1) and the topological consistency (Theorem 3.4) deal with violations pointed out by Wang^40^, and the approximation operators to interior/closure operations, respectively, by Lashin et al.^42^.

### Practical implications

Photovoltaic fault detection case study confirms the practical applicability HFSRS is better to the classical methods in all measures (Table 5). The 7% point increase compared to classical RS and the 35% decrease in the boundary area translate to a smaller number of false alarms in industrial monitoring. Renewable energy technology choice proves to be discriminating in decision-making situations of complexity.

### Limitations

While synthetic data provides rigorous validation under controlled conditions with known ground truth, it may not capture all complexities of actual PV system behavior including infrequent fault patterns, environmental interactions, and sensor drift. The β selection is domain-specific and requires calibration for new applications. The $\:O(n\times\:m\times\:k)$ complexity may limit scalability when k (average hesitant values) is large. Future work should validate on real operational data through collaboration with energy operators.

## Conclusion and future scope

This article established the HFSRS framework addressing major shortcomings in uncertainty modeling through novel integration of hesitant fuzzy soft sets and rough sets. Dynamic $\:\beta\:$ coverings represent a paradigm shift from static approximation boundaries to adaptive thresholds responding to uncertainty levels. Theoretical contributions include provable duality preservation (Theorem 3.1), monotonicity (Theorem 3.2), and hesitant fuzzy topological foundations (Theorem 3.4) connecting approximation operators to interior/closure operations.

Empirical validation demonstrated superior performance with 92% accuracy compared to 85% for Classical RS, 88% for IFS-RS, and 86% for Fuzzy RS. Statistical significance was confirmed through paired t-tests (*p* < 0.001) and ROC analysis (AUC = 0.97). The optimal $\:\beta\:$ threshold of 0.65 provides practical guidance for threshold selection, balancing 92% accuracy with 8% boundary region.

The HFSRS framework establishes foundations for next-generation decision support systems in domains characterized by multi-dimensional uncertainty including renewable energy planning, industrial diagnostics, and healthcare. Future research directions include: (1) validation on real-world operational data from solar farms and industrial plants, (2) extension to interval-valued hesitant fuzzy environments, (3) integration with machine learning algorithms for adaptive threshold optimization, and (4) development of distributed computing implementations for big data applications.

## Data Availability

The data and code supporting the findings of this study are openly available on GitHub at https://github.com/Jahanvi-Phd/HFSRS.git The repository includes the HFSRS framework implementation, datasets used in the case studies, and scripts to reproduce all results presented in this paper.
